# Rapamycin retards epigenetic ageing of keratinocytes independently of its effects on replicative senescence, proliferation and differentiation

**DOI:** 10.18632/aging.101976

**Published:** 2019-05-26

**Authors:** Steve Horvath, Ake T. Lu, Howard Cohen, Ken Raj

**Affiliations:** 1 Human Genetics, David Geffen School of Medicine, University of California, Los Angeles, Los Angeles, CA 90095, USA; 2 Biostatistics, Fielding School of Public Health, University of California, Los Angeles, Los Angeles, CA 90095, USA; 3 Elizabeth House Medical Practice, Warlingham, Surrey CR6 9LF, United Kingdom; 4 Radiation Effects Department, Centre for Radiation, Chemical and Environmental Hazards, Public Health England, Chilton, Didcot, Oxfordshire OX11 0RQ, United Kingdom

**Keywords:** epigenetic ageing, rapamycin

## Abstract

The advent of epigenetic clocks has prompted questions about the place of epigenetic ageing within the current understanding of ageing biology. It was hitherto unclear whether epigenetic ageing represents a distinct mode of ageing or a manifestation of a known characteristic of ageing. We report here that epigenetic ageing is not affected by replicative senescence, telomere length, somatic cell differentiation, cellular proliferation rate or frequency. It is instead retarded by rapamycin, the potent inhibitor of the mTOR complex which governs many pathways relating to cellular metabolism. Rapamycin, however, is also an effective inhibitor of cellular senescence. Hence cellular metabolism underlies two independent arms of ageing – cellular senescence and epigenetic ageing. The demonstration that a compound that targets metabolism can slow epigenetic ageing provides a long-awaited point-of-entry into elucidating the molecular pathways that underpin the latter. Lastly, we report here an *in vitro* assay, validated in humans, that recapitulates human epigenetic ageing that can be used to investigate and identify potential interventions that can inhibit or retard it.

## Introduction

One of the biggest challenges in ageing research is the means of measuring age independently of time. This need becomes particularly clear when we wish to evaluate the effects of drugs or compounds on ageing, where the use of time as a measure of age is clearly inappropriate. In recent years, several age-estimators known as epigenetic clocks have been developed, which are based on methylation states of specific CpGs, some of which become increasingly methylated, while others decreasingly so with age [1]. Age estimated by these clocks is referred to as epigenetic age or more precisely, DNA methylation age (DNAm age). The “ticking” of these clocks is constituted by methylation changes that occur at specific CpGs of the genome. Significantly, the increased rate by which these specific methylation changes occur is associated with many age-related health conditions [1–9], indicating that epigenetic clocks, capture biological ageing (epigenetic ageing) at least to some extent. The numerous epigenetic clocks that have been independently developed [10–16] differ in accuracy, biological interpretation and applicability, whereby some epigenetic clocks are compatible only to some tissues such as blood. In this regard, the pan-tissue epigenetic clock [2] stands out because it is applicable to virtually all tissues of the body, with the exception of sperm. It estimates the same epigenetic age for different post-mortem tissues (except the cerebellum and female breast) from the same individual [2,8]. Although the pan-tissue epigenetic clock performs extremely well with *in vivo* cell samples, its accuracy was not as good with fibroblasts and other *in vitro* cell samples. We addressed this recently by developing an even more accurate multi-tissue age estimator, which we refer to as skin & blood clock [3], which is applicable for *in vivo* as well as *in vitro* samples of human fibroblasts, keratinocytes, buccal cells, blood cells, saliva and endothelial cells. *In vitro* human cell culture systems offer many advantages including tight control of growth conditions, nutrients, cell proliferation rates, detailed morphological analyses and genetic manipulation, all of which are impractical or inappropriate in human cohort studies. Hence the availability of an *in vivo* epigenetic clock, such as the skin & blood clock that can also be used for *in vitro* experiments is an important and significant step towards uncovering the molecular mechanisms that underpin epigenetic ageing.

Although the molecular mechanisms of epigenetic ageing remain largely uncharacterised, the cellular aspects however, have been explored to a greater albeit limited degree. The similar epigenetic ages detected amongst different tissue of the same body [2,8] suggests that epigenetic age is not a measure of cellular proliferation since the rate and frequency of proliferation differ greatly between different tissues such as blood, which is highly proliferative and heart cells, which are post-mitotic. It is intuitive to make a connection between epigenetic ageing and senescent cells, which increases in number with age and which mediates phenotypic ageing [8,17]. This attractive link, however, was discounted by previous reports which clearly excluded DNA damage, telomere attrition and cellular senescence as drivers of epigenetic aging [18].

A way to further characterise epigenetic ageing is through the evaluation of validated anti-aging interventions on it. Such an intervention is the nutrient response pathway regulated by the mammalian target of rapamycin (mTOR) [19–21]. Although originally developed as an immunosuppressant, rapamycin has emerged as one of the most impressive life-extending compounds [22]. It has been repeatedly shown to extend the lives of different animal species including those of yeast [23], flies [24] and mice [25,26]. The structure of rapamycin presents two major sites for potential interactions. The binding of one site to FKBP12 protein, allows its other site to bind and inhibit the mTOR kinase [27]. This kinase is part of a complex that promotes cell growth, proliferation and cell survival [28,29]. This may be why mTOR activity is often elevated in cancer cells; the rationale behind its use as an anti-cancer drug [30]. By inhibiting mTOR activity, rapamycin also recapitulates to some extent, the effect of calorie-restriction, which has also been repeatedly shown to prolong the lives of many different animal species [31]. As such, rapamycin is widely considered to be a promising anti-ageing intervention. Here we characterise epigenetic aging in primary human keratinocytes from multiple donors by testing their sensitivities to rapamycin and we observed that it can indeed mitigate epigenetic ageing independently of cellular senescence, proliferation, differentiation and telomere elongation.

## RESULTS

### Opposing effects of Rapamycin and ROCK inhibitor on keratinocyte proliferation

**Figure 1:**
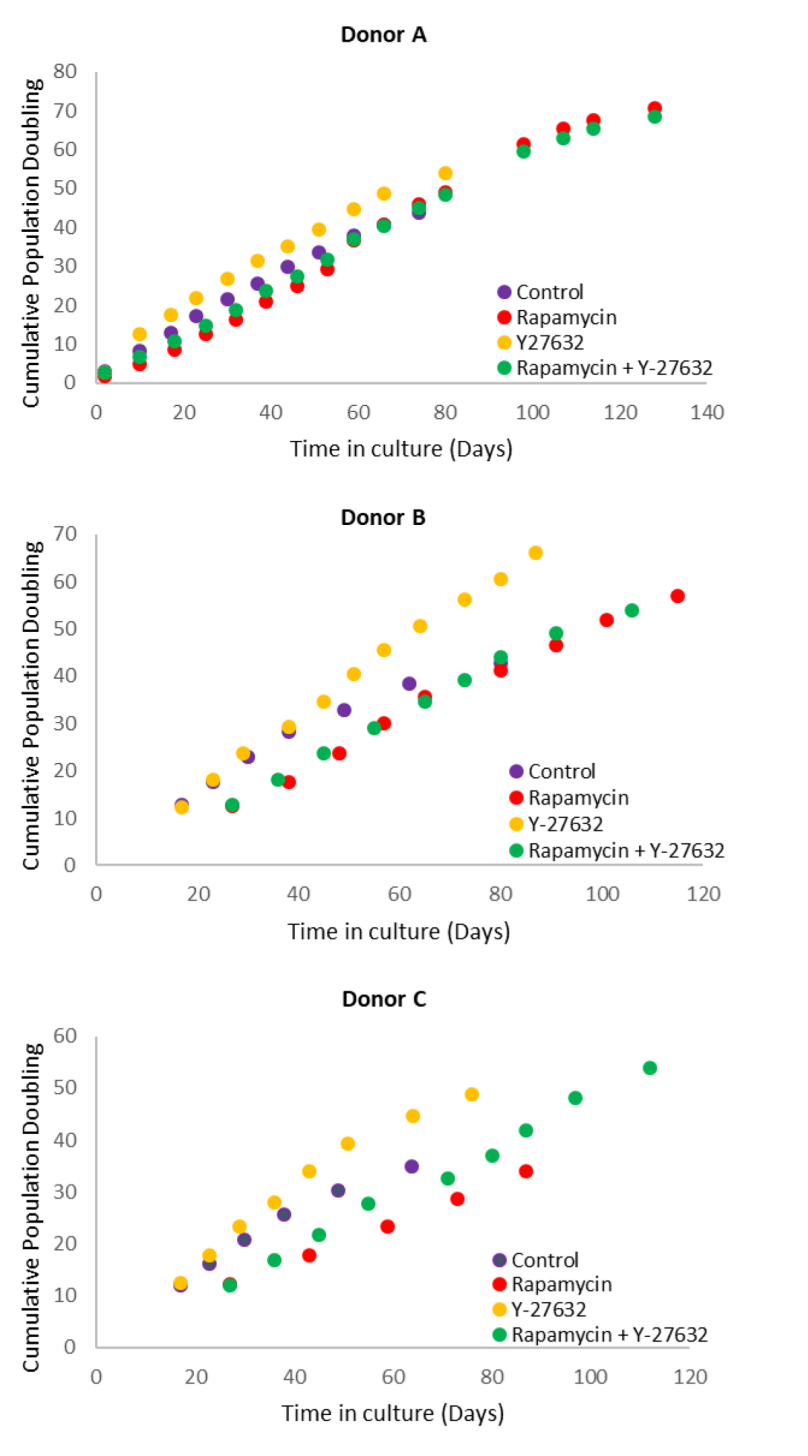
**Effects of rapamycin and Y-27632 on the proliferation of keratinocytes from Donors A, B and C that were used in the subsequent experiments.** Cells from Donors A, B and C were cultured in the continued presence of the indicated compounds. Population doubling at every cell passage was ascertained until replicative senescence, and plotted against time.

The availability of an epigenetic clock, such as the skin & blood clock, which is applicable to cultured cells, allows epigenetic ageing to be studied beyond the purely descriptive nature afforded by epidemiological analyses alone. Towards this end, we have established *in vitro* epigenetic ageing systems using primary human cells. One of this is based on primary keratinocytes that are derived from healthy human skins. As previously reported by others, we observed that the proliferation rate of these cells, which is defined as the number of population doublings per unit of time, can be significantly altered by different compounds. Rapamycin, which is the primary focus of this investigation reduces cellular proliferation rate, while Y-27632, which inhibits Rho kinase (ROCK inhibitor) increases it, and a mixture of both modestly alleviates the repressive effect of rapamycin (Figure 1 and Table 1). The opposing effects of these compounds on keratinocyte proliferation present us with the opportunity to test whether cellular proliferation rate impacts epigenetic ageing while carrying out our primary aim of interrogating the effects of rapamycin on epigenetic ageing.

**Table 1:** Population doubling times (in hours) of primary neonatal foreskin keratinocytes from three donors (A, B and C) cultured in various conditions.

**Culture condition**	**Donor A**	**Donor B**	**Donor C**
**Control**	38.1	38.2	41.6
**Rapamycin**	42.8	46.5	66.0
**Y‐27632**	30.1	31.6	30.3
**Rapamycin + Y‐27632**	39.6	44.5	49.1

### Effects of Rapamycin and Y-27632 on epigenetic ageing

**Figure 2:**
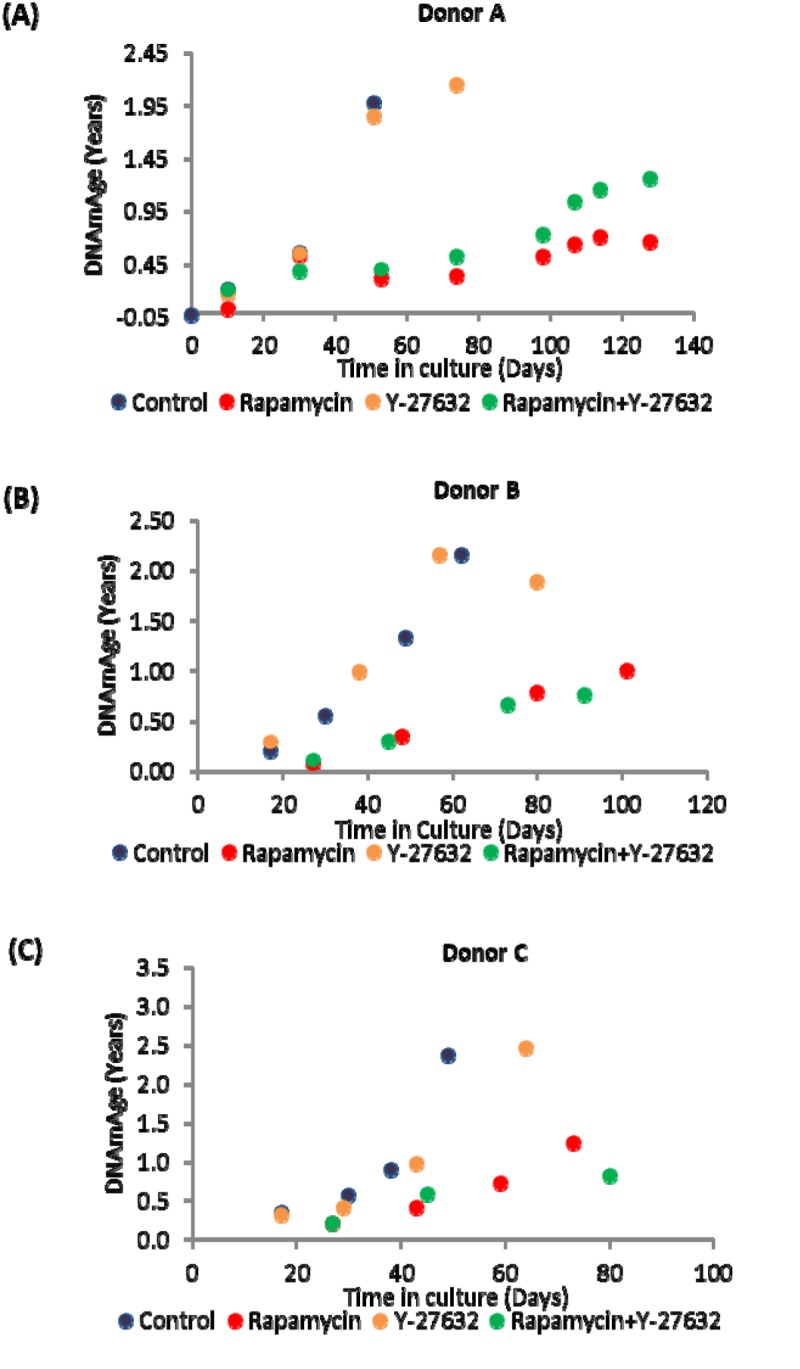
**Ageing dynamics of keratinocytes of (A) Donor A, (B) Donor B and (C) Donor C in the presence or absence of rapamycin and Y-27632.** Methylation profiles of DNA from selected passages of each cell population were analysed and their ages estimated with the skin and blood clock. The colour allocated to each culture condition is preserved throughout for ease of comparison.

Primary keratinocytes were isolated from human neonatal foreskins from three donors (Donor A, B and C) and were put in culture with standard media or media supplemented with rapamycin, Y-27632 or a cocktail of both of these compounds (methods). The cells were passaged continually and population doublings at each passage recorded. In time all cells, regardless of donor or treatment underwent replicative senescence, where they ceased to increase their numbers after at least 2 weeks in culture with regular replenishment of media. Interestingly, two of the three donor cells treated with rapamycin underwent further proliferation before replicative senescence, indicating that their proliferative capacity was increased (Figure 1 and Table 2). This was also observed with Y-27632-treated cells. DNA methylation profiles from a selection of passages of these cells were obtained and analysed with the skin & blood clock. It is clear from figure 2 that while Y-27632 did not impose any appreciable effect, rapamycin retarded epigenetic ageing of these cells. This is evident even when Y-27632 was present with rapamycin. These empirical observations demonstrate three fundamental features of epigenetic ageing. First, increased cellular proliferation rate, as instigated by Y-27632 (Figure 1 and Table 1) does not affect epigenetic ageing. This echoes the conclusion derived from analyses of *in vivo* tissues, using the pan-tissue age estimator [2] and confirmed by Yang et al. [32] who specifically derived a DNA methylation-based mitotic clock to be able to measure cellular proliferation, as epigenetic ageing clocks were not able to do so. Second, increased proliferative capacity (the number of times cells proliferate before replicative senescence) is not inextricably linked with retardation of epigenetic ageing since rapamycin and Y-27632 can both instigate the former, but only rapamycin-treated cells exhibited retardation of epigenetic ageing. Third, epigenetic ageing is not a measure of replicative senescence since all rapamycin-treated cells eventually underwent replicative senescence and yet remained younger than the un-treated control cells; an observation that would not be made were epigenetic age a measure of senescent cells.

**Table 2:** Cumulative population doubling of keratinocyte cultures from three donors (A, B and C) at the point of replicative senescence.

**Culture condition**	**Donor A**	**Donor B**	**Donor C**
**Control**	44	43	35
**Rapamycin**	71	57	34
**Y‐27632**	54	66	49
**Rapamycin + Y‐27632**	69	54	54

### Somatic cell differentiation does not drive epigenetic ageing

**Figure 3:**
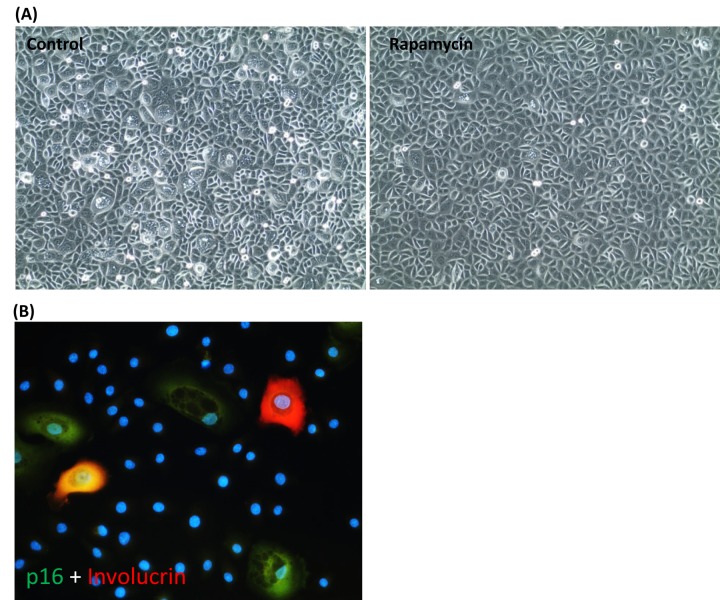
**Rapamycin suppresses the emergence of senescent cells and spontaneous keratinocyte differentiation.** (**A**) Phase contrast picture of primary keratinocytes cultured in CnT-07 medium in the absence (left panel) or presence (right panel) of rapamycin. (**B**) The large keratinocytes seen in **(A**) were stained positive with antibodies against p16 (in green), involucrin (red) or both, p16 and involucrin (yellow). Nuclei were stained with DAPI.

Having ruled out cellular proliferation rate and proliferation capacity, as well as replicative senescence as drivers of epigenetic ageing, we considered the possible role of somatic cell differentiation in this regard. We observed that healthy primary keratinocytes in culture are heterogeneous in size and shape, but those that were growing in the presence of rapamycin were much more regular in shape and have considerably fewer enlarged cells (Figure 3A). Staining with antibodies against p16; a marker of senescent cells [33], and involucrin; a marker of early keratinocyte differentiation [34], showed that the enlarged cells were a mixture of senescent cells and differentiating cells, with some cells exhibiting both markers (Figure 3B). As our previous investigations [18] and observations above have uncoupled cellular senescence from epigenetic ageing, we questioned whether cellular differentiation could instead be the driver and the ability of rapamycin to reduce spontaneous differentiation may be the way by which it retards epigenetic ageing.

**Figure 4:**
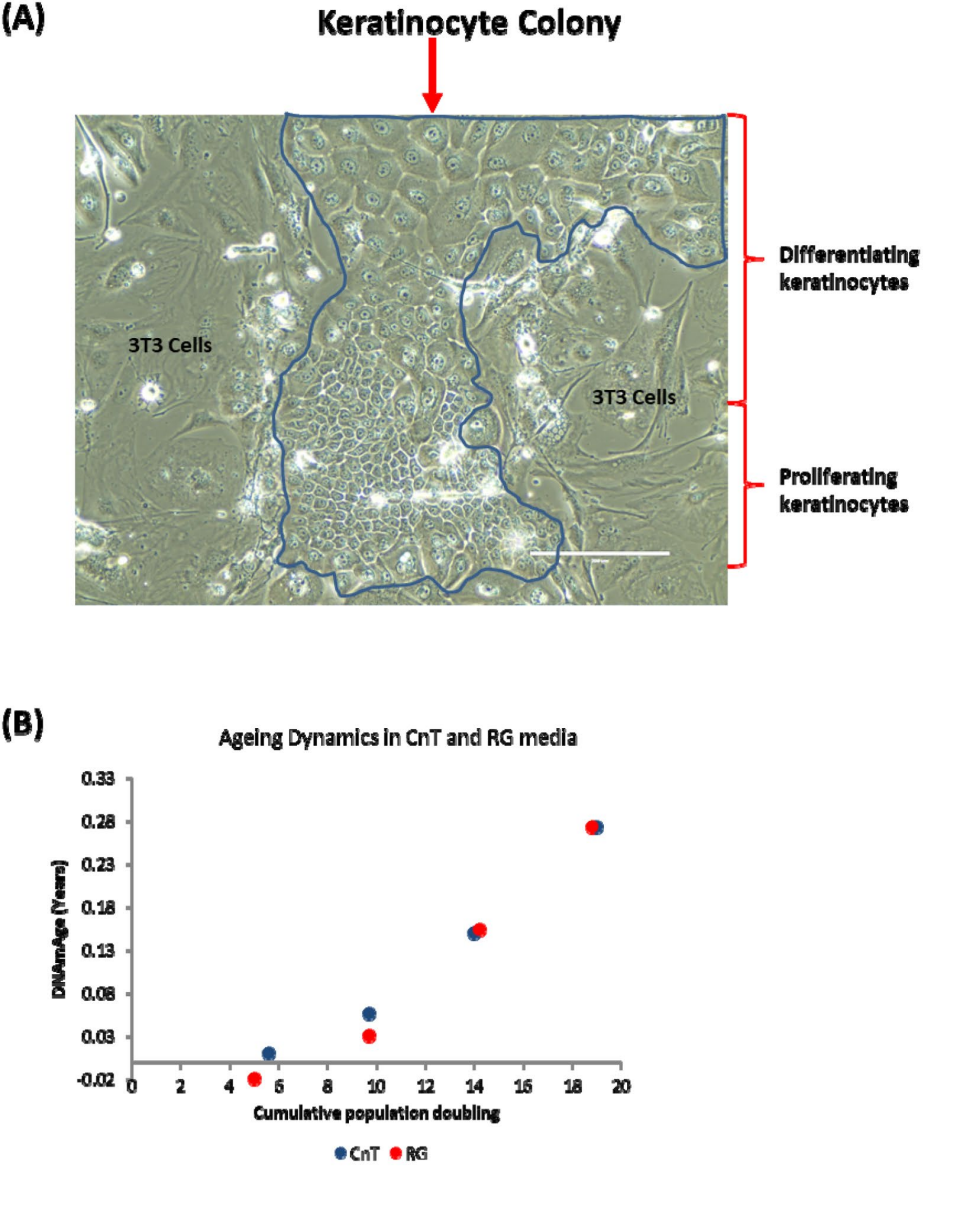
**Keratinocyte differentiation does not drive epigenetic ageing.** (**A**) Phase contrast image of a primary keratinocyte colony grown in the presence of irradiated J2-3T3 feeder cells in RG medium. The keratinocyte colony is demarcated within the blue boarder and proliferating or differentiating keratinocytes are indicated. Cells external of the boarder are irradiated 3T3-J2 feeder cells. (**B**) Comparison of epigenetic aging between primary keratinocytes grown in CnT-07 media (CnT) and RG media (RG).

In the experiments described thus far, primary keratinocytes were grown in a culture condition where the medium used (CnT-07) was designed with the expressed purpose of encouraging the proliferation of progenitor keratinocytes, while restricting their spontaneous differentiation; evidently not eliminating it altogether. To test the hypothesis that cellular differentiation drives epigenetic ageing, we opted to encourage spontaneous keratinocyte differentiation to see if this would cause a rise in their epigenetic age. To this end, we cultured human primary keratinocytes in a different medium, as reported by Rheinwald and Green [35], and with mouse 3T3 cells, which serve as feeder cells. Crucially, this culture condition which we term RG not only supports the proliferation of keratinocytes, it also permitted spontaneous differentiation to a much greater extent than does CnT media. Figure 4A shows a typical keratinocyte colony grown in RG condition. The colony is constituted by small proliferating cells as well as considerable number of large cells in different stages of differentiation.

Primary keratinocytes from the same human donor (Donor D) were cultured in these two different conditions described above (CnT and RG). DNA methylation profiles from four passages of cells, with known number of population doubling were obtained and their ages were estimated by the skin & blood clock. Figure 4B shows that encouraging greater keratinocyte differentiation by culturing them in RG condition did not increase epigenetic ageing, demonstrating that contrary to the hypothesis, epigenetic ageing is not increased by greater keratinocyte differentiation and therefore the retardation of epigenetic ageing by rapamycin is not mediated through its suppression of spontaneous somatic cell differentiation. Collectively, these experiments have demonstrated that rapamycin is an effective retardant of epigenetic ageing, and that this activity is mediated independently of its effects on replicative senescence and somatic cell differentiation.

## DISCUSSION

It is widely assumed that extension of lifespan is a result of retardation of ageing. While there is no counter-evidence to challenge this highly intuitive association, supporting empirical evidence to confirm it is not easy to acquire. As a case in point, improvement in public health in the past century has extended life-span, but there is no directly measurable evidence that this was accompanied by a reduction in the rate of ageing. The same question could be asked of any intervention that purports to extend life. The scarcity of empirical evidence is due in part to the lack of a good measure of age that is not based on time. In this regard, the relatively recent development of epigenetic clocks is of great interest [1]. Despite their impressive performance, almost nothing is known about the molecular components and pathways that underpin them. At the cellular level however, more is known, but from the perspective of what epigenetic ageing is not, rather than what it is. The bringing together of rapamycin and the skin & blood clock in the experiments above have shed light on both of them. This has been significantly enhanced by comparison with the effects, or not, of the Rho kinase inhibitor, Y-27632. As a case in point, the retardation of epigenetic ageing by rapamycin could have been erroneously ascribed to the retardation of the rate of keratinocyte proliferation, were it not for the fact that Y-27632 augments proliferation rate but does not increase epigenetic ageing. This precludes a simplistic and incorrect correlation between the rate of cellular proliferation and epigenetic ageing. Recently Yang et al demonstrated that epigenetic ageing clock tracks cellular proliferation very poorly compared to the purpose-built DNA methylation-based mitotic clock [32].

The impulse to turn our attention and ascribe retardation of epigenetic ageing to reduced senescent cells is understandable since rapamycin does indeed reduce the emergence of these cells in cultures, as consistent with previous characterisation and description [36–41]. This notion however is inconsistent with our previous finding that the epigenetic age of a cellular population is not dependent on the presence of senescent cells [18], and this conclusion is further confirmed here, where all the rapamycin-treated cells eventually senesced, without any rise in their epigenetic age. Therefore, while rapamycin’s inhibition of senescence is not in doubt, this is not the means by which it retards the progression of epigenetic age of keratinocytes.

To test whether somatic cell differentiation drives epigenetic ageing, we refrained from using chemical means to induce terminal differentiation of keratinocytes as this could introduce DNA methylation changes that might confound interpretation of the results. Instead, we exploited the propensity of keratinocytes to spontaneously differentiate, which they do significantly better in RG medium than in CnT-07 medium [42]. The hypothesis that differentiation drives epigenetic ageing was clearly refuted by these observations. While we still do not know what cellular feature is associated with epigenetic ageing, we can now remove somatic cell differentiation from the list of possibilities and place it with cellular senescence, proliferation and telomere length maintenance, which represent cellular features that are all not linked to epigenetic ageing.

The ability of rapamycin to suppress the progression of epigenetic ageing is very encouraging for many reasons not least because it provides a valuable point-of-entry into molecular pathways that are potentially associated with it. Evidently, the target of rapamycin, the mTOR complex is of particular interest. It acts to promote many processes including, but not limited to protein synthesis, autophagy, lipid synthesis and glycolysis [29,43,44]. The experiments above were not designed to identify the specific mTOR activity or activities that underpin epigenetic ageing, but they point to further experiments involving gene manipulation and drugs that could be brought to address this question. It is of great significance that we have previously identified through genome-wide association studies (GWAS), genetic variants near MLST8 coding region whose expression levels are positively correlated with epigenetic aging rates in human cerebellum [45]. MLST8 is a subunit of the mTORC1 and mTORC2 complexes, and its gene expression levels increase with chronological age in multiple brain regions [45]. It is pivotal for mTOR function as its deletion prevents the formation of mTORC1 and mTORC2 complexes [46]. The convergence of the GWAS observation with the experimental system described here is a testament of the strength of the skin & blood clock in uncovering biological features that are consistent between the human level and cellular level. It lends weight to the emerging view that the mTOR pathway may be the underlying mechanism that supports epigenetic ageing.

It is of interest to note that the experimental set-up above constitutes an *in vitro* ageing assay that is applicable not only to pure research but to screening and discovering other compounds and treatments that may mitigate or suppress epigenetic ageing. Most biological models of human diseases or conditions are derived from molecular, cellular or animal systems that rightly require rigorous validation in humans. In this regard, the epigenetic clock is distinct in being derived from, and validated at the human level. Hence *in vitro* experimental observations made with it carry a significant level of relevance and can be readily compared with an already available collection of human data generated by the epigenetic clock – the MSLT8 described above is an example in point. An added advantage of such a validated *in vitro* ageing system for human cells is the ability to test the efficacy of potential mitigators of ageing in a well-controlled manner, within a relatively short time, at a significantly low cost and with the ability to ascertain whether the effects are on life-span, ageing or both; all of which are not readily achieved with human cohort studies.

We wish to acknowledge some limitations inherent in this investigation. First, we have not tested this activity of rapamycin on all cell types and we caution the reader that interventions against epigenetic aging can be cell-type specific: for example, menopausal hormone therapy appears to slow epigenetic ageing of buccal cells (which are predominantly keratinocytes) but not that of blood [47]. Second, while we have used primary keratinocytes derived from numerous donors, they were all from neonatal tissues. This is a necessary constrain at this early stage of the investigation in order to avoid confounding effects of age. It would be necessary to test the efficacy of rapamycin on adult donors across the entire age spectrum (0-100 years). Finally, it is important to note that it is inadvisable (actively discouraged) to directly extrapolate the studies here, especially in terms of the magnitude of age suppression, to potential effects of rapamycin on humans.

In summary, the observations above represent the first biological connection between epigenetic ageing and rapamycin. These results for human cells add to the evidence that extension of life, at least by rapamycin, is indeed accompanied by retardation of ageing. These observations also suggest that the life-extending property of rapamycin may be a resultant of its multiple actions which include, but not necessarily limited to suppression of cellular senescence [36–38,48] and epigenetic aging, with the possibility of augmentation of cellular proliferative potential.

## MATERIALS AND METHODS

### *In vitro* cultured cell procedure

### *Isolation and culture of primary keratinocytes*

Primary human neonatal fibroblasts were isolated from circumcised foreskins. Informed consent was obtained prior to collection of human skin samples with approval from the Oxford Research Ethics Committee; reference 10/H0605/1. The tissue was cut into small pieces and digested overnight at 4 °C with 0.5 mg/ml Liberase DH in CnT-07 keratinocyte medium (CellnTech) supplemented with penicillin/streptomycin (Sigma) and gentamycin/amphotericin (Life Tech). Following digestion, the epidermis was peeled off from the tissue pieces and placed in 1 millilitre (ml) of trypsin-versene. After approximately 5 minutes of physical desegregation with forceps, 4 ml of soybean trypsin inhibitor was added to the cell suspension and transferred into a tube for centrifugation at 1,200 revolutions per minute for 5 minutes. The cell pellet was resuspended in CnT-07 media and seeded into fibronectin/collagen-coated plates. Cells were grown at 37 °C, with 5% CO_2_ in a humidified incubator. Growth medium was changed every other day. Upon confluence, cells were trypsinised, counted and 100,000 were seeded into fresh fibronectin/collagen-coated plates. Population doubling was calculate using the following formula: [Log(number of harvested cells)- log(number of seeded cells)] X 3.32. Rapamycin was used at 25nM and Y-27632 at 1μM concentrations and were present in the media of treated cells for the entire duration of the experiments. RG medium was prepared by mixing three parts of F12 medium with one part DMEM, supplemented with 5% foetal calf serum, 0.4ug/ml hydrocortisone, 8.4ng/ml cholera toxin, 5ug/ml insulin, 24ug/ml adenine and 10ng/ml epidermal growth factor. 3T3-J2 cells were cultured in DMEM supplemented with 10% foetal calf serum. To prepare feeder cells, 3T3-J2 cells were irradiated at 60Gy and seeded onto fibronectin/collagen-coated plates in RG medium at least 6 hours but no more than 24 hours prior to seeding of keratinocytes. To harvest keratinocytes grown in RG media, feeder cells were first removed with squirting of the monolayer with trypsin-versene for approximately 3 minutes, after which the monolayer was rinsed with 7ml of Phosphate Buffered Saline (PBS) followed by incubation of the monolayer with 0.5ml of trypsin-versene. When all the keratinocytes have lifted off the plate, 1ml of soybean trypsin inhibitor was added to the cell suspension. Cells were counted and 100,000 were seeded into fresh plates as described above.

### *Immunofluorescence*

Cells were grown on glass coverslips that were pre-coated with fibronectin-collagen. When ready, the cells were fixed with formalin for 10 minutes, followed by three rinses with Phosphate Buffered Saline (PBS). Cell membranes were permeabilised with 0.5% TritonX-100 for 15 minutes followed by three 5 minute rinses with PBS. Primary antibodies diluted in 2% foetal calf serum in PBS were added to the cells. After 1 hour the antibodies were removed followed by three 5 minute rinsing, after which secondary antibodies (diluted in 2% foetal calf serum in PBS) was added. After 30minutes, the antibodies were removed and the cells were rinsed five times with 1ml PBS each time for five minutes followed by a final rinse in 1 ml distilled water before mounting on glass slide with Vectastain. Cells were imaged using a fluorescence microscope. Antibodies used were as follows: Anti-Involucrin (Abcam ab53112) diluted at 1:1000 and Anti-p16 (Bethyl laboratories A303-930A-T) diluted at 1:500.

### *DNA methylation studies and epigenetic clock*

DNA was extracted from cells using the Zymo Quick DNA mini-prep plus kit (D4069) according to the manufacturer’s instructions and DNA methylation levels were measured on Illumina 850 EPIC arrays according to the manufacturer’s instructions. The Illumina BeadChips (EPIC or 450K) measures bisulfite-conversion-based, single-CpG resolution DNAm levels at different CpG sites in the human genome. These data were generated by following the standard protocol of Illumina methylation assays, which quantifies methylation levels by the β value using the ratio of intensities between methylated and un-methylated alleles. Specifically, the β value is calculated from the intensity of the methylated (M corresponding to signal A) and un-methylated (U corresponding to signal B) alleles, as the ratio of fluorescent signals β = Max(M,0)/[Max(M,0)+ Max(U,0)+100]. Thus, β values range from 0 (completely un-methylated) to 1 (completely methylated). We used the "noob" normalization method, which is implemented in the "minfi" R package [49,50]. The mathematical algorithm and available software underlying the skin & blood clock (based on 391 CpGs) is presented in Horvath et al., 2018 [3].
